# Modifier Effects between Regulatory and Protein-Coding Variation

**DOI:** 10.1371/journal.pgen.1000244

**Published:** 2008-10-31

**Authors:** Antigone S. Dimas, Barbara E. Stranger, Claude Beazley, Robert D. Finn, Catherine E. Ingle, Matthew S. Forrest, Matthew E. Ritchie, Panos Deloukas, Simon Tavaré, Emmanouil T. Dermitzakis

**Affiliations:** 1 Wellcome Trust Sanger Institute, Wellcome Trust Genome Campus, Hinxton, United Kingdom; 2 Department of Oncology, University of Cambridge and Cancer Research UK Cambridge Research Institute, Li Ka Shing Centre, Cambridge, United Kingdom; The University of Queensland, Australia

## Abstract

Genome-wide associations have shown a lot of promise in dissecting the genetics of complex traits in humans with single variants, yet a large fraction of the genetic effects is still unaccounted for. Analyzing genetic interactions between variants (epistasis) is one of the potential ways forward. We investigated the abundance and functional impact of a specific type of epistasis, namely the interaction between regulatory and protein-coding variants. Using genotype and gene expression data from the 210 unrelated individuals of the original four HapMap populations, we have explored the combined effects of regulatory and protein-coding single nucleotide polymorphisms (SNPs). We predict that about 18% (1,502 out of 8,233 nsSNPs) of protein-coding variants are differentially expressed among individuals and demonstrate that regulatory variants can modify the functional effect of a coding variant in *cis*. Furthermore, we show that such interactions in *cis* can affect the expression of downstream targets of the gene containing the protein-coding SNP. In this way, a *cis* interaction between regulatory and protein-coding variants has a *trans* impact on gene expression. Given the abundance of both types of variants in human populations, we propose that joint consideration of regulatory and protein-coding variants may reveal additional genetic effects underlying complex traits and disease and may shed light on causes of differential penetrance of known disease variants.

## Author Summary

The ultimate goal of genome-wide association studies (GWAS) is to explain the proportion of variation in a phenotypic trait that can be attributed to genetic factors. The past two years have seen a plethora of successes in this field, yet, for most traits, a large fraction of variation remains unexplained. Epistasis, or interaction between genetic variants, is a largely under-explored factor, which may shed some light in this area. We use the HapMap populations to investigate interactions between regulatory and protein-coding variants and their impact on gene expression. We show that if a specific protein-coding variant has a functional impact, this can be modified by a co-segregating regulatory variant (*cis* interaction). Furthermore, the authors demonstrate that such modification effects between variants at one locus may affect the expression of other genes in the cell in a *trans* manner. The aim of this article is to present a framework though which variation can be considered in the context of GWAS. Viewing variation from this underappreciated angle may, in some cases, provide an explanation for differential penetrance of complex disease traits, but also for non-replication of GWAS results that may arise as a consequence of such interactions.

## Introduction

Most disease association studies to date attempt to link single genetic variants to a specific phenotype [1],[2],[3],[4]. The genetic interaction between variants, also called epistasis, results in a phenotypic effect that is conditional on the combined presence of two or more variants [5],[6]. The prevalence and biological significance of epistasis has always been an area of interest in the field of human genetics and quantitative genetics, but its contribution to phenotypic variation has remained obscure, largely because genetic interactions have proven difficult to test [7]. This difficulty arises primarily because it is unclear which variant combinations should be tested and under which model of epistasis. To date, most strategies that address the effects of epistasis in humans involve millions of agnostic pairwise tests and fall into two broad categories: exhaustive testing of interactions between all pairs of variants across the genome [8], or testing of interactions between all pairs of those variants that each have an independent main effect on the phenotype of interest [8],[9],[10]. It is not entirely clear whether improvements in statistical methods will be sufficient to address the problem of epistasis. Therefore, the development of realistic biological models of epistatic interactions may reduce the statistical cost of dealing with many comparisons and facilitate the development of such methodologies.

To date, such an approach has been most feasible in model organisms and for specific genes or biological pathways that have been well-characterised. One classic example is the *Adh* locus in Drosophila where a series of regulatory SNPs in complex linkage disequilibrium (LD) modify the effects of a protein-coding variant [11],[12]. The protein-coding variant affects the catalytic efficiency of the ADH protein, whereas the regulatory variants have an impact on protein concentration. Catalytic efficiency and protein levels affect the overall activity of ADH showing that large effects attributed to a single locus may arise as a consequence of multiple associated variants. More recent studies in Drosophila reveal epistatic effects between genes affecting traits such as ovariole number [13] and olfactory avoidance [14]. In cases where little is known about the genes sculpting a phenotype, addressing the possibility of epistasis becomes more challenging. The value of assessing the impact of genetic interactions is highlighted in a recent study interrogating cardiac dysfunction in Drosophila [15]. A major susceptibility locus for this trait has been detected, but the importance of examining the phenotype in different genetic backgrounds was highlighted as a means to detect variants contributing to the phenotype through interactions with the prime susceptibility locus. The extent of epistasis in a more global way has been demonstrated in yeast where experiments on gene expression revealed that interacting locus pairs are involved in the inheritance of over half of all transcripts[5]. Furthermore, a large proportion of the eQTLs attributable to interaction effects were not detected by single locus tests. This suggests that analysis of interaction effects in other systems is likely to uncover additional associations.

In humans, most documented cases of epistasis have been detected in instances where there are biological clues as to which genes should be tested for interaction. Epistasis between two multiple sclerosis (MS) associated HLA-DR alleles was demonstrated by Gregerson et al. [16] who showed that one allele modifies the T-cell response activated by a second allele, through activation-induced apoptosis contributing to a milder form of MS-like disease. Similarly, Oprea et al. [17] demonstrated that a specific modifier effect is protective against spinal muscular atrophy (SMA). SMA arises from a homozygous deletion of *SMN1*, but some deletion homozygotes escape the disease phenotype due to the modulating effects of expression of *PLS2*. With the explosion of successful genome-wide association studies over the past two years, the need to address epistasis in a systematic, genome-wide approach is becoming increasingly pressing. The case of MS clearly illustrates this: as with most complex disorders, MS has a polygenic heritable component characterised by underlying complex genetic architecture [18]. Association studies to date have met with modest success in identifying MS-causing genes, and a large proportion of phenotypic variation remains unexplained. The expectation is that this residual variation arises at least in part, as a consequence of gene-gene interactions. Finally, epistasis may mask and prevent replication of otherwise real genetic effects due to differential fixation of variants that modulate the primary disease variant and affect the degree of penetrance of certain disease alleles.

In this study we propose a biological framework that could be useful for global survey of epistatic (modifier) effects in humans, which avoids exhaustive testing of agnostic pairs and involves prioritisation of variants to be tested. Two types of functional variants are common throughout the human genome and are present at appreciable frequencies in populations: regulatory variants with an impact on the expression patterns and levels of genes [19],[20],[21],[22],[23] and protein-coding nucleotide variants affecting protein sequence [19],[24]. To date, the effects of these variants have been considered independently of each other. In this study we perform an evaluation of the joint effects of regulatory and protein-coding variants to genome-wide expression phenotypes in humans in order to highlight an underappreciated angle of functional variation.

## Results

### The Model of Epistasis

**Figure 1:**
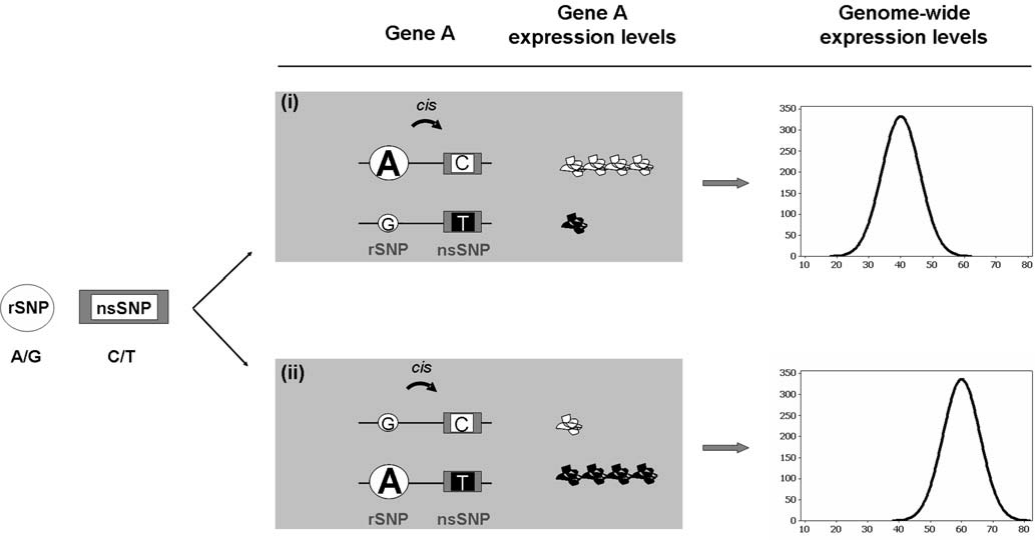
Illustration of a hypothetical epistatic interaction between a regulatory and a protein-coding variant. Two double heterozygote individuals may be genotypically identical, but the phasing of alleles can be different and may result in very distinct phenotypes between individuals. In one individual (i) the A allele of the rSNP drives high expression levels of the protein arising from the C allele of the nsSNP. In another individual (ii) the G allele of the rSNP drives low expression levels of the protein arising from the C allele of the nsSNP. If the protein-coding variant is functionally important then this can give rise to different means in the distribution of a complex trait phenotype as shown on the right.

Our model brings together quantitative and qualitative variation. A gene that has an identified *cis* regulatory variant is differentially expressed among individuals of a population where that variant is segregating [20],[23]. If this gene also contains coding variation, then, assuming that mRNA levels are indicative of mature protein levels, the resulting protein products will not only differ in quantity (expression level) but also in quality or type (amino acid sequence) among individuals. Furthermore, depending on the historical rate of recombination between the regulatory and the coding variants, different allelic combinations (haplotypes) can arise on the two homologous chromosomes in a population. Phasing, the arrangement of the alleles at each variant with respect to one another, can differ between individuals in the population (Figure 1) [25]. If this is the case, the epistasic effect arising from these two variant types can be explored under a specific and testable biological model.

Using this model as a main principle, we explored the degree to which protein-coding variants can be modulated by *cis*-acting regulatory variants in human populations. In a previous study [20] we identified a set of SNPs (minor allele frequency (MAF)≥0.05) implicated in regulation of activity of genes in EBV-transformed lymphoblastoid cell lines (LCLs) of the unrelated individuals of the HapMap populations [26],[27] (60 Caucasians of Northern and Western European origin (CEU), 45 unrelated Chinese individuals from Beijing University (CHB), 45 unrelated Japanese individuals from Tokyo (JPT), and 60 Yoruba from Ibadan, Nigeria (YRI)). LCLs represent one particular cell type and even though there may be some effect arising from EBV transformation, it has been demonstrated that genetic effects on gene expression , such as the ones we describe below, are readily identifiable and mappable, and replicate in independent population samples. We henceforth refer to genetic variants associated with gene expression levels as candidate regulatory SNPs (rSNPs) and regard them as proxies for the linked functional variants that drive differential expression levels of nearby genes. The protein-coding variants considered under this model are non-synonymous SNPs (nsSNPs), i.e. variants that give rise to an amino acid substitution in the protein product. nsSNPs harboured in genes with varying expression levels are hereon termed differentially expressed (DE).

### Differentially Expressed nsSNPs

Two strategies were applied to detect DE nsSNPs in the HapMap populations. The first strategy involved scanning genes with known rSNPs [20] for nsSNPs. The aim was to identify nsSNPs that are predicted to be DE as a consequence of a nearby regulatory variant tagged by an identified rSNP. From the 606, 634, 679 and 742 genes with rSNPs previously identified [20] (0.01 permutation threshold and estimated false discovery rate (FDR) = 20%) in the CEU, CHB, JPT and YRI respectively, we found 159, 168, 180 and 202 of these genes (union of 484) containing 286, 304, 311 and 393 nsSNPs respectively (union of 909) (Table S1). We infer that these nsSNPs are DE as they reside in genes with experimentally-derived varying expression levels. This means that there are allelic effects on gene expression such that, depending on the genotypes of the rSNP and nsSNP and on the phasing of their alleles, one can make predictions about the relative abundance of the two alleles of a transcript in the cell.

The second strategy for DE nsSNP discovery involved direct association testing between nsSNP genotype and expression levels of the gene in which the nsSNP resides. We performed the test for each expressed gene harbouring at least one nsSNP. This strategy aimed to identify DE nsSNPs that are in LD with a regulatory variant. Depending on the strength of the regulatory effect, such variants may or may have not been detected in our initial scan for rSNPs. Relative distances between rSNPs and nsSNPs can vary, but in the special case where this distance is short in genetic terms, the two variants may be in LD [25]. Under these circumstances we expect that the nsSNP itself will demonstrate some degree of association with expression levels of the gene in which it resides. We used standard methodologies described in Stranger *et al.* 2007 [20] (see Methods) to test for genotype-expression associations in each population and in three multiple-population sample panels: (a) all four HapMap populations, (b) three populations (CEU-CHB-JPT), and (c) two populations (CHB-JPT). The choice of these panels represents a pooling strategy by which we sequentially remove individuals of the most genetically distant population sample.

For the single-populations analysis, with significance evaluated at the 0.01 significance threshold as determined by 10,000 permutations, we expect 56 nsSNPs and 34 genes to have at least one significant association by chance. We detected 242, 276, 267 and 255 nsSNPs (union of 703; estimated FDR ∼21%) with significant associations to expression levels of the gene in which they reside for the CEU, CHB, JPT and YRI populations respectively. These associated nsSNPs correspond to 196, 226, 210 and 211 genes (union of 560; estimated FDR ∼16%) (Table 1). For the multiple-population analysis at the same significance threshold (using conditional permutations that account for population differentiation–see Methods), we detected 345, 362 and 417 nsSNPs (estimated FDR ∼15%) for the four, three and two population groups respectively, corresponding to 284, 296 and 320 significant genes (estimated FDR ∼11%) (Table 1). Overall, the multiple-population analysis yielded a total of 587 nsSNPs with significant associations, corresponding to 461 genes. Taken together, the association analyses indicate that 884 nsSNPs (688 genes) are associated with expression levels of the genes they are in, suggesting that they are in LD with regulatory variants driving their expression. In this specific case of association, the nsSNP itself serves as a proxy to the regulatory variant. Therefore, knowledge of associated nsSNP genotype for an individual enables us to make a prediction about the relative abundance of the two alleles of a transcript containing the nsSNP.

**Table 1:** nsSNP and gene *cis* associations in single and multiple-population subsets.

	0.01 permutation threshold						
	1	2	3	4			
Population	significant nsSNPs	CEU-CHB-JPT-YRI multipop	CEU-CHB-JPT multipop	CHB-JPT multipop	Overlap 1&2	Overlap 1&3	Overlap 1&4
**CEU**	242	345	362	417	111	139	104
**CHB**	276	345	362	417	126	162	224
**JPT**	267	345	362	417	136	161	203
**YRI**	255	345	362	417	102	86	90
**Nonredundant**	703						
**4 populations**	34						
**≥2 populations**	233						

	0.01 permutation threshold						
	1	2	3	4			
Population	significant genes	CEU-CHB-JPT-YRI multipop	CEU-CHB-JPT multipop	CHB-JPT multipop	Overlap 1&2	Overlap 1&3	Overlap 1&4
**CEU**	196	284	296	320	99	117	87
**CHB**	226	284	296	320	109	129	183
**JPT**	210	284	296	320	114	125	156
**YRI**	211	284	296	320	87	77	82
**Nonredundant**	560						
**4 populations**	31						
**≥2 populations**	196						

**Figure 2:**
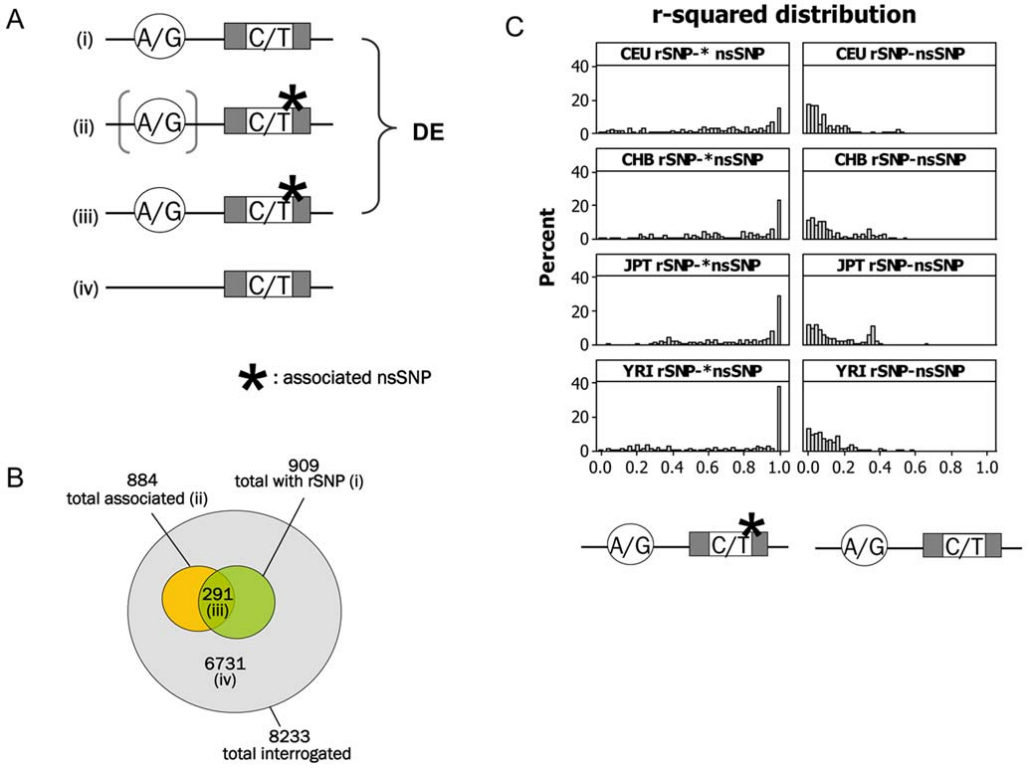
Strategies applied to discover differentially expressed (DE) nsSNPs and linkage disequilibrium properties between rSNP-nsSNP pairs. (A) Two approaches were employed to discover DE nsSNPs: nsSNPs mapping in genes with a known rSNP (i) and nsSNPs that were associated with expression levels of the gene they map in (ii). In (ii) the presence of a *cis*-acting regulatory variant is implied. For some nsSNPs with a significant association, an identified *cis* rSNPs also exists (iii). In all other cases the nsSNPs interrogated were not inferred to be DE (iv). (B) (i) 909 nsSNPs map in a gene with an identified rSNP; (ii) 884 nsSNPs were found to be associated with levels of gene expression of the gene they reside in; (iii) the overlap between i and ii (nsSNPs with an identified rSNP that also showed a significant association) is 291 (iii). 6731 nsSNPs show no evidence for DE. (C) The distribution of r-squared (a measure of LD) was compared between rSNP-nsSNP pairs in which the nsSNP showed a significant association (at the 0.01 permutation threshold) and SNP pairs in which the nsSNP was not associated. As expected, r-squared values are much higher in the first case, in which the nsSNP is thought to act as a tag of the functional regulatory variant nearby.

To summarize, two classes of DE nsSNPs were discovered: (a) nsSNPs mapping in genes with a previously-identified rSNP (909 nsSNPs, considering nsSNPs of all frequencies) and (b) nsSNPs showing a significant association with expression levels of the gene they are in (884 nsSNPs, considering nsSNPs with MAF≥0.05) (Figure 2a & b). From a non-redundant total of 8233 nsSNPs tested, we predict that 1502 of these (∼18.2%) are DE. If mature protein levels mirror on average transcript levels, which is a reasonable biological hypothesis, then this high fraction has important implications for the levels of protein diversity in the cell.

### Linkage Disequilibrium between rSNPs and nsSNPs

Of the 884 DE nsSNPs discovered through association testing (set b above), only 291 also possess a previously identified rSNP. This suggests that rSNP detection in our previous study [20] was conservative and that nsSNPs can act as tags of (markers for) nearby, undiscovered regulatory variants. With this in mind, we expect that LD between rSNP- nsSNP pairs in which the nsSNP had a significant association (0.01 permutation threshold) with gene expression, will be greater than LD between rSNP- nsSNP pairs in which the nsSNP was not associated. To explore this, we used data from the single population analysis, and compared the distribution of r-squared values (a measure of LD) between the two rSNP-nsSNP pair types. As expected, we found much higher LD between rSNP-nsSNP pairs where the nsSNP showed a significant association (Mann-Whitney p<0.0001) (Figure 2c). This confirms that in most cases, association of the nsSNP with gene expression of its own gene is due to a regulatory variant that acts as proxy to the identified rSNP.

### Experimental Verification of Differentially Expressed nsSNPs

So far we have used genotypic associations, not direct allele-specific quantification (allele-specific expression; ASE), to derive relative abundance estimates for transcripts of genes containing nsSNPs. To experimentally verify the predictions of the association tests (i.e. that the alleles of associated nsSNP are DE), we tested a subset of nsSNPs for allele-specific expression [22],[28] in heterozygote CEU and YRI individuals. The initial experiment included a total of 141 nsSNPs from category (b) of DE nsSNPs. nsSNPs of this category provide a prediction of the relative abundance of the two alleles as transcripts in the cell. The assay performed was new and proved noisy. As a result it was possible to confirm and analyze signals for 28 nsSNPs. For individuals heterozygous for each nsSNP, we assigned relative expression of the two alleles. We then compared the experimentally derived relative abundance, by ASE, with the predictions of relative abundance from the genotypic association test. We found that predicted and experimentally-quantified relative expression of alleles of nsSNPs were in agreement for 89% (16 out of 18) and 90% (9 out of 10) of nsSNPs tested in the CEU and the YRI populations, respectively. This is in agreement with the FDR estimated above. This strongly suggests that the relative abundance of alternative coding transcripts can be inferred reliably by genotypic associations.

### Properties of Differentially Expressed nsSNPs

To assess the potential biological impact of DE nsSNPs we compared three functional attributes of those amino acid substitutions arising from DE nsSNPs and those arising from non-DE nsSNPs (nsSNP MAF≥0.05, to assess common nsSNP consequences). We investigated: (1) the relative position of substitution on the peptide, as different effects may arise depending on whether the nsSNP is at the beginning or the end of the peptide), (2) the resulting change in peptide hydrophobicity which may alter the interactions of a protein [29], and (3) the resulting change in Pfam score (a measure of amino acid profile in each position of a protein domain)[30], which assesses the integrity of protein domains that are evolutionary conserved and likely to harbour important functions. In all cases the properties of DE nsSNPs were not different from those of nonDE nsSNPs. Though indirect and not comprehensive, this signal suggests that DE nsSNPs may be a random subset of nsSNPs (Figure S1 a–c).

To assess how many DE nsSNPs have a known function, we explored the OMIM database [31] and found that 71 (out of 1502) DE nsSNPs have an OMIM entry (Table S2). DE nsSNPs were found to map in genes with a role in cancer susceptibility (*BRAC1* (+113705), *BARD1* (+113705)), asthma and obesity (*ADRB2* (+109690)), Crohn's disease (*DLG5* (*604090)), myokymia (*KCNA1* (*176260)), diabetes (*OAS1* (*164350)), chronic lymphatic leukaemia (*P2RX7* (*602566)) emphysema and liver disease (*P I*(+107400)), severe keratoderma (*DSP* (+125647)), and familial hypercholesterolemia (*ABCA1* (+600046)). In some cases the functional role of the nsSNP remains unclear and the noise in reported functional effects in OMIM is well-known and very difficult to assess in a study such as the present, but there are examples where specific effects have been attributed to nsSNPs. For example, rs28931610 in *DSP* is predicted to change disulphide bonding patterns and alter the peptide tertiary structure; rs28933383 in *KCNA1* causes a substitution in a highly conserved position of the potassium channel and is predicted to impair neuronal repolarization; rs28937574 in *P2RX7* is a loss of function mutation associated with chronic lymphatic leukaemia; rs28931572 in *PI* entails a replacement of a polar for a non-polar amino acid and is predicted to disrupt tertiary structure of the protein, and rs2230806 in *ABCA1* is associated with protection against coronary heart disease in familial hypercholesterolemia. The modulation of such strong effects by *cis* regulatory variation may increase the complexity and severity of these biological effects.

### Genetic Interaction between rSNP and nsSNP

**Figure 3:**
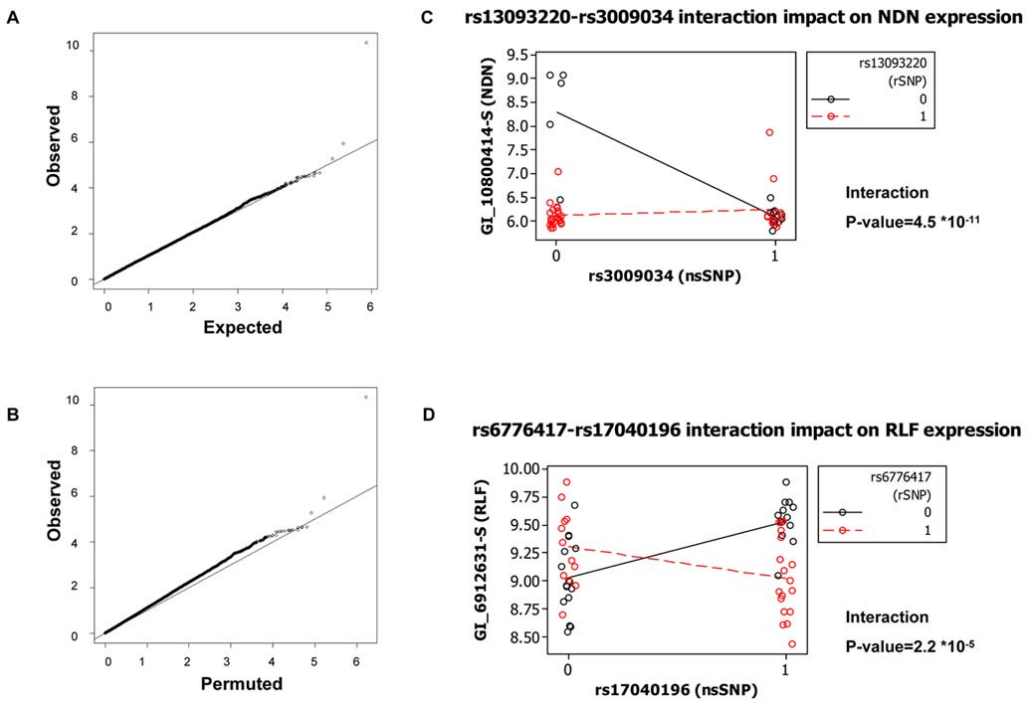
Impact of rSNP-nsSNP genetic interaction on *trans* gene expression. (A) QQ plot of observed –log_10_pvalues of the interaction term in the ANOVA over expected (under the assumption of a Uniform distribution of p-values). (B) QQ plot of observed –log_10_pvalues of the interaction term in the ANOVA over the –log_10_pvalues of the interaction term in the permuted data. (C) Example 1: The interaction between rs13093220 (rSNP) and rs3009034 (nsSNP) on chromosome 3, is associated with changes in expression of *NDN* (probe ID GI_10800414-S) on chromosome 15 (interaction p = 4.5*10^−11^). (D) Example 2: The interaction between rs6776417 (rSNP) rs17040196 (nsSNP) on chromosome 3 is associated with changes in expression of *RLF* (probe ID GI_6912631-S) on chromosome 1 (interaction p = 2.2*10^−5^).

Thus far we have presented indirect evidence for an interaction in *cis* where the effect of an nsSNP is modulated by a co-segregating regulatory variant tagged by an rSNP. Under such circumstances, and if the gene containing the nsSNP has downstream targets, then it is likely that the expression of downstream genes may also be affected. In other words, apart from the modification effect observed in *cis*, we wanted to test for the genome-wide effects of this interaction directly, in a statistical framework. To do this we carried out ANOVA by testing the main effects of rSNPs and nsSNPs and their interaction term (rSNP×nsSNP) on genome-wide gene expression (*trans* effects). The rationale behind this approach is that if an rSNP-nsSNP interaction is biologically relevant, its effect may influence the expression of downstream targets of the gene harbouring the rSNP-nsSNP pair. The power to detect an interaction is maximized when all combinations of genotypes are present, each at appreciable frequencies in the population. To increase power of interaction detection, we pooled rare homozygotes with heterozygotes into a single genotypic category, creating a 2×2 table of genotypes. This does not bias our statistic as shown by permutations below. We performed this analysis in the CEU population sample as CHB and JPT population samples were small (45 individuals) and the YRI sample has generally shown low levels of *trans* effects in previous analyses [20]. We tested 22 rSNP-nsSNP pairs (SNP pairs) with low LD (D′≤0.5) and a MAF≥0.1 for both SNPs, against genome-wide expression. At the 0.001 nominal p-value threshold, we expect roughly 331 significant associations (assuming a uniform distribution of p-values) for the interaction term. We observe 412, which corresponds to an estimated FDR of 80%. This is overall a weak signal (see also Figure 3a), but signals at the tail of the distribution appear to be real given the limited power of this analysis (Figure 3c, d).

To test for potential biases in the statistic used, we carried out the same tests using permuted gene expression values (a single permutation was done by maintaining the correlation structure of gene expression data–see Methods) relative to the rSNP-nsSNP genotypes. We explored the p-value distribution of the rSNP-nsSNP interaction for observed and permuted data (Figure 3b) and found an abundance of low p-values in the observed data. There appears to be some degree of p-value inflation in the observed data relative to the permuted data which is most likely due to correlations in gene expression data. This however does not affect the enrichment of p-values seen in the tails of the observed distribution relative to expected distributions (uniform distribution of p-values) or the p-value distributions derived from permuted gene expression data. The permutation was not done in order to assess significance thresholds but rather in order to assess the enrichment of tests with low p-values in the observed data and is in agreement with the enrichment derived from the enrichment under a uniform distribution of p-values. To further evaluate the robustness of the interactions, we repeated the analysis for the top 10 rSNP-nsSNP significant pairs against their corresponding *trans*-associated gene expression phenotype, after permuting rSNP genotypes relative to nsSNP genotypes and gene expression values. As expected, the significance of the interaction vanishes in the permuted data. The conditional effects of alleles at the rSNP and nsSNP loci can therefore have a very different impact on the expression of other genes in the cell. This conditional effect on gene expression is illustrated in Figures 3 c and d which show two examples of an rSNP-nsSNP interaction (p = 4.5×10^−11^ and p = 2.2×10^−5^), and in Table 2 where the summary statistics and specific information of SNPs and genes are illustrated for the 10 most significant interaction effects. These plots illustrate the effect on gene expression of different rSNP-nsSNP genotypic combinations. In Figure 3c for example, SNP rs3009034 has an effect on gene expression of gene *NDN* only if the genotype of SNP rs13093220 is homozygous for the common allele. The phenotypic effect of such interactions is even more prominent in Figure 3d where we observe opposite directions of the effect of SNP rs1704196 on gene *RLF* depending on the genotype on SNP rs6776417.

**Table 2:** The pairs of SNPs with the 10 most significant interaction effects in *trans* with summary statistics and information about mapping location and genes they derive from and affect.

rSNP	nsSNP	source Refseq	source chr	Refseq start	rSNP loc	rSNP MAF	nsSNP loc	nsSNP MAF	nsSNP aa subst	trans gene Hugo	trans chr	rSNP pval	nsSNP pval	Interaction pval
rs13093220	rs3009034	NM_001039757	3	75861803	75853445	0.453	75864268	0.242		NDN	15	1.04E-04	3.41E-05	4.56E-11
rs2280902	rs2272720	NM_003970	8	1980655	2064479	0.433	2008828	0.467	V/L	TFF2	21	1.88E-02	3.35E-03	1.20E-06
rs2280902	rs2272720	NM_003970	8	1980655	2064479	0.433	2008828	0.467	V/L	Hs.525661	15	1.03E-01	1.16E-02	5.41E-06
rs6776417	rs17040196	NM_032137	3	14691658	14674018	0.267	14720861	0.35	A/T	RLF	1	1.46E-01	2.89E-01	2.22E-05
rs6776417	rs6790129	NM_032137	3	14691658	14674018	0.267	14730621	0.342	L/V	RLF	1	1.46E-01	2.89E-01	2.22E-05
rs6776417	rs17040196	NM_032137	3	14691658	14674018	0.267	14720861	0.35	A/T	C6orf57	6	3.40E-01	8.91E-02	2.36E-05
rs6776417	rs6790129	NM_032137	3	14691658	14674018	0.267	14730621	0.342	L/V	C6orf57	6	3.40E-01	8.91E-02	2.36E-05
rs6776417	rs17040196	NM_032137	3	14691658	14674018	0.267	14720861	0.35	A/T	Hs.121413	2	8.84E-01	8.96E-01	3.10E-05
rs6776417	rs6790129	NM_032137	3	14691658	14674018	0.267	14730621	0.342	L/V	Hs.121413	2	8.84E-01	8.96E-01	3.10E-05
rs2280902	rs2272720	NM_003970	8	1980655	2064479	0.433	2008828	0.467	V/L	hmm11130	2	3.51E-01	6.33E-02	3.17E-05

## Discussion

We have presented a biological framework to interrogate functional genetic variation by focusing on a specific case of epistasis between regulatory and protein-coding variants. We have shown that regulatory variants may have an impact on the protein diversity of cells by differentially modulating the expression of protein-coding variants. In *cis*, regulatory variants can amplify or mask the functional effects of protein-coding variants, which might consequently result in a milder or more severe phenotype to the one expected if only the protein-coding variant were present. We have shown that such interactions can affect the expression of other genes in the cell (*trans* effect), in a manner that can only be revealed if the interaction term of the two variants is considered.

The conditional effects of alleles of functional variants may therefore have important consequences for complex phenotypic traits. The extent to which epistasis affects phenotypes remains an under-explored area, but the critical importance of such interactions is starting to emerge [17]. We provide a biological framework for considering and conditioning existing disease associations on known regulatory and protein-coding variants, in an approach that also provides a potential explanation for the differential penetrance of known disease variants. The abundance of *cis* regulatory and protein-coding variants in human populations and the generic nature of this type of epistatic interaction (no assumptions about specific biological pathways) makes it very likely that such interactions are common genetic factors underlying complex traits and their consideration is likely to reveal important associations that have not been detected thus far. Furthermore, this consideration is particularly important for studies that fail to replicate the primary disease associations in newly tested populations, since it is hypothesized that some of the failures are due to differential frequency of modifier alleles between the first and second population. The consideration of the interactions described above may assist in better interpretation of non-replicated signals.

## Methods

### Gene Expression Quantification and Normalization

Total RNA was extracted from lymphoblastoid cell lines of the 210 unrelated individuals of the HapMap [26],[27] (Coriell, Camden, New Jersey, United States). Gene expression (mRNA levels) was quantified using Illumina's commercial whole genome expression array, Sentrix Human-6 Expression BeadChip version 1 (∼48,000 transcripts interrogated; Illumina, San Diego, California, United States) [32]. Hybridization intensity values were normalized on a log_2_ scale using a quantile normalization method [33] across all replicates of a single individual followed by a median normalization method across all 210 individuals. A subset of 14,456 probes (13,643 unique autosomal genes) that were highly variable within and between populations was selected from the 47,294 probes on the array, and were used for the analysis. A detailed description can be found in Stranger *et al.* [20].

### Nonsynonymous (nsSNPs) and Regulatory (rSNPs) SNPs

HapMap nsSNPs (version 21, NCBI Build 35) were mapped onto Refseq genes using nsSNP and gene coordinate information. rSNPs are defined as those phase II HapMap SNPs (version 21, NCBI Build 35, minor allele frequency (MAF)≥0.05) with a *cis* significant association at the 0.01 permutation threshold, as described in Stranger *et al.* [20]. The genomic location of rSNPs is within 1 Mb from the probe genomic midpoint.

### nsSNP Association Analyses

We tested nsSNP genotype for association with expression levels of the gene it is in using an additive linear regression model [3],[20],[34] applied to each population separately. Our association analysis employed: 1) nsSNP genotypes for the unrelated individuals of each HapMap population (MAF≥0.05) from the HapMap phase II map for each population (version 21, NCBI Build 35) and 2) normalized log_2_ quantitative gene expression measurements for the 210 unrelated individuals of each of the original four HapMap populations (60 Caucasians of Northern and Western European origin (CEU), 45 unrelated Chinese individuals from Beijing University (CHB), 45 unrelated Japanese individuals from Tokyo (JPT), and 60 Yoruba from Ibadan, Nigeria (YRI)).

### nsSNP Association and Multiple-Test Correction Single Population Analysis

To assess significance of association between nsSNP genotype and expression variation of the gene harbouring the nsSNP, we performed 10,000 permutations of each expression phenotype relative to the genotypes [20]. An association to gene expression was considered significant if the nominal p-value from the linear regression test was lower than the 0.01 tail of the distribution of the minimal p-values (among all comparisons for a given gene) from each of the 10,000 permutations of the expression phenotypes. For genes containing more than one nsSNP the most stringent permuted p-value was retained.

### Multiple Population Panels

To increase the power of the nsSNP association analysis we combined data (nsSNP genotypes and normalized expression values) for unrelated individuals of multiple populations [20]. We compiled three different multiple population comparison panels: 1) CEU-CHB-JPT-YRI, 2) CEU-CHB-JPT, 3) CHB-JPT. Association tests were carried out for each population panel separately using linear regression. Conditional permutations (randomization of data within each population as described in Stranger et al. [20]were performed to assess significance of the nominal p-values. This approach accounts for the population differentiation and prevents detection of spurious associations [20]. For each of the 14,456 probes in each multiple population panel, expression values were permuted among individuals of a single population followed by regression analysis of the grouped multi-population expression data against the grouped multi-population permuted nsSNP genotypes. Associations were considered significant if the nominal p-value was lower than the threshold of the 0.01 tail of the distribution of the minimal p-values from the 10,000 permutations of the expression phenotypes. For genes containing more than one nsSNP the most stringent permuted p-value was retained. It is important to note that in all cases permutations maintained the correlated structure of gene expression values (i.e. all gene expression values were randomized as a block for each individual).

### DNA and RNA Preparation for Allele-Specific Expression (ASE) Assay

Genomic DNA (gDNA) and total RNA were extracted from lymphoblastoid cell lines of the unrelated CEU and YRI HapMap individuals (Coriell, Camden, New Jersey, United States) using Qiagen's AllPrep kit. RNA was treated with Turbo DNA-free (Ambion) to minimize gDNA contamination. The RNA was concentrated and further cleaned with RNeasy MinElute columns (Qiagen). Total RNA and gDNA were quantified using Nanodrop Spectrophotometer and either Quant-iT RNA or DNA reagents (Invitrogen). Double stranded (ds) cDNA was synthesised from 250 ng of cleaned RNA. The first strand was synthesised with Superscript III (Invitrogen) and random hexamers. The second strand was synthesised with DNA polymerase I (Invitrogen), ribonuclease H (Invitrogen) and dNTPs. The 96-well plate containing the ds cDNA samples was cleaned using Multiscreen PCR plate (Millipore).

### Illumina ASE Array

The Oligo Pool All (OPA) was custom made by illumina and is based on the Golden Gate assay. Only exonic SNPs≥45 bp from both exon edges were chosen for submission to illumina for assay design to ensure that the assay would work equally well for genomic and cDNA. SNPs that failed according to illumina's design scores were discarded. Paired ds cDNA and gDNA were dried down in 96-well plates and re-suspended in 5 µl of HPLC purified water. Golden Gate assays were then run for all samples using the manufacturer's standard protocol for gDNA (i.e. ds cDNA was treated exactly the same way as gDNA). Reactions were hybridised to 8×12 Sentrix Array Matrix (SAM) Universal Probe Sets so that 96 arrays could be run in parallel. Each bead type (probe) is present on a single array on average 30 times. All reactions were run in duplicate, so that each cell line had two ds cDNA replicate and two gDNA replicate hybridizations. SAMs were scanned with a Bead Station (illumina). A total of 1536 assays were interrogated on the array but only 141 were nsSNPs from this study and only 28 were selected based on data quality for further analysis.

### ASE Assay Data Pre-Processing

Data from each array was summarised by calculating the per bead type average of 4 quantities after outlier removal: the log_2_(Cy3) and log_2_(Cy5) intensities, average log-intensities (^1^/_2_log_2_(Cy5.Cy3)) and log-ratios (log_2_(Cy5/Cy3)). Outliers were beads with values more than 3 median absolute deviations from the median. Arrays with low dynamic range (determined using an inter-quartile range cut-off of less than 1 for either the log_2_(Cy3) or log_2_(Cy5) summary intensities) were discarded. The summarised data was normalized by median centering the log-ratios. All analysis was carried out in R using the beadarray package [35]. Direction of expression (high/low) was assigned to alleles for nsSNPs fulfilling threshold criteria from the association study (adjusted r^2^ value≥0.27; i.e. the nsSNP explained at least 27% of the variance in gene expression of the gene it is found in so the effect is expected to be large) and the ASE assay (average cDNA log-intensity≥12 within a population).

### Assignment of Differentially Expressed (DE) nsSNPs

In each population an nsSNP is defined to be DE if: 1) it maps within a gene that also has an independently identified *cis* rSNP or 2) it shows a significant association with its own gene's expression levels. We tested those rSNPs with the strongest association per gene with nsSNPs of all frequencies. The total number of nsSNPs that are predicted to be DE is the non-redundant union of 1) and 2).

### rSNP-nsSNP Pair Linkage Disequilibrium (LD) Analysis

LD values (r-squared and D′) were calculated by a pairwise estimation between rSNPs and nsSNPs genotyped in the same individuals and within a 100 kb window (Ensembl version 46). LD estimates for rSNP-nsSNP pairs with and without an associated nsSNP (0.01 permutation threshold) were compared using a Mann-Whitney U test.

### Amino Acid Substitution Effect

Given that nsSNPs are likely to be functional we explored three aspects of the resulting amino acid substitution: 1) Relative position of substitution on the peptide, as a percent of peptide total length. 2) Hydrophobicity change in peptide resulting from the amino acid substitution. For each pair of variant sequences the hydrophobicity at the position of the variant amino acid was calculated using the Kyte-Doolittle algorithm using a window size of 7 (centred on the variant amino acid). The difference between hydrophobicty scores was then taken for each of the variant pairs in the dataset. 3) Pfam score change in peptide sequence resulting from the amino acid substitution. All sequences were searched against the profile-HMM library provided by the Pfam database (release 22.0) using hmmpfam from the HMMer software package (version 2.3.2, http://hmmer.janelia.org/) and a default cut off E-value of 10. Only the HMM_ls library was used so that domain assignments to a pair of variant sequences were comparable. The set of Pfam domain assignments were then filtered such that only the domains that overlapped with the SNP position and that at least one of the domain assignments from a pair of variant sequences scored above the Pfam defined gathering threshold, were considered in the subsequent analysis. The difference between the two E-values was then taken for each of the variant pairs in the dataset.

### *Trans* Association Analysis

Our aim was to test the interaction effects of rSNP with nsSNP on expression phenotypes in *trans* in the CEU population Our strategy involved pooling the minor allele homozygote and the heterozygote into a single genotypic category and then coding genotypes as 0 (major allele homozygote) or 1 (heterozygote and minor allele homozygote) for both SNP types. As a result four possible rSNP-nsSNP genotypic combinations were possible: 0-0, 1-0, 0-1, 1-1. We performed ANOVA to test the effects of the rSNP, the nsSNP, and the interaction term (rSNP×nsSNP) in the same model against gene expression phenotypes in *trans* (in each case excluding the gene from which the rSNP-nsSNP pair originates). Tests were carried out for 22 SNP pairs with low LD (D′≤0.5) between the rSNP and the nsSNP and a MAF≥0.1 for both variants (to avoid outlier effects).

To assess significance of the interaction p-values we generated a single permuted dataset of expression values relative to the combined genotypes and compared the p-value distribution of the interaction term for the observed and the permuted data. To further evaluate the robustness of the observed interactions we permuted the rSNP genotypes relative to nsSNP genotypes and gene expression phenotypes, and re-ran the ANOVA association test for the top 10 most significant interactions.

## Supporting Information

Figure S1 We compared three functional attributes between amino acid substitutions arising from differentially expressed (DE) nsSNPs and those arising from non-DE nsSNPs. We investigated: (A) the relative position of substitution on the peptide, (B) the resulting change in peptide hydrophobicity [29] and (C) the resulting change in Pfam score when searched against the Pfam profile Hidden Markov Model library [30]. We conclude that DE nsSNPs appear to be a random subset of nsSNPs. Therefore, if a random nsSNP has a phenotypic effect, this is likely to be amplified or masked through differential expression caused by a *cis*-acting regulatory variant.
(0.91 MB TIF)

Table S1 nsSNPs and genes interrogated for differential expression.
(0.01 MB PDF)

Table S2 Differentially expressed nsSNPs in OMIM.
(0.02 MB PDF)
