# Supplementary figures and images for: Modifier Effects between Regulatory and Protein-Coding Variation
Source: PLoS Genet. 2008 Oct 31;4(10):e1000244. doi: 10.1371/journal.pgen.1000244 (PMC2570624; doi:10.1371/journal.pgen.1000244)

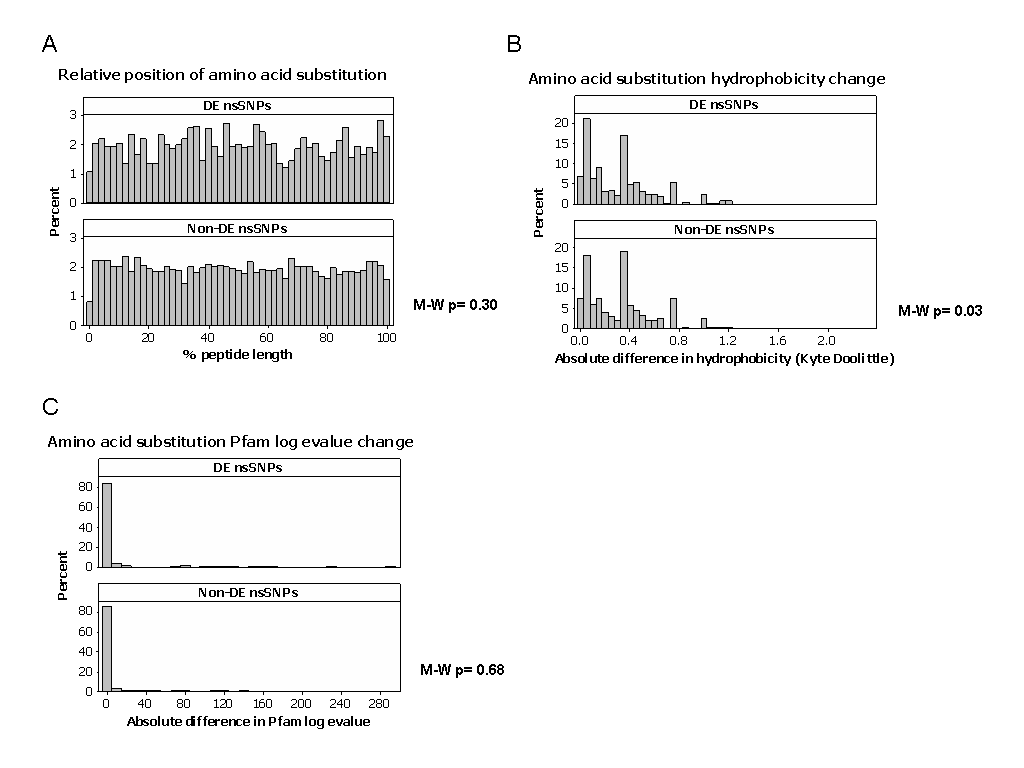

Supplement: Figure S1 — We compared three functional attributes between amino acid substitutions arising from differentially expressed (DE) nsSNPs and those arising from non-DE nsSNPs. We investigated: (A) the relative position of substitution on the peptide, (B) the resulting change in peptide hydrophobicity [29] and (C) the resulting change in Pfam score when searched against the Pfam profile Hidden Markov Model library [30]. We conclude that DE nsSNPs appear to be a random subset of nsSNPs. Therefore, if a random nsSNP has a phenotypic effect, this is likely to be amplified or masked through differential expression caused by a cis-acting regulatory variant. (0.91 MB TIF) [file pgen.1000244.s001.tif]
