# Supplementary material for: Modifier Effects between Regulatory and Protein-Coding Variation
Source: PLoS Genet. 2008 Oct 31;4(10):e1000244. doi: 10.1371/journal.pgen.1000244 (PMC2570624; doi:10.1371/journal.pgen.1000244)
Supplement: Table S1 — nsSNPs and genes interrogated for differential expression. (0.01 MB PDF) [file pgen.1000244.s002.pdf]

Table S1

	nsSNPs						genes					
	**All nsSNPs interrogated**	**with rSNP**	**asc-single pop**	**single pop DE**	**asc-multi-pop**	**All DE**	**All genes interrogated**	**with rSNP**	**asc-single pop**	**single pop DE**	**asc-multi-pop**	**All DE**
CEU	5686	286	242	452			3579	159	196	307		
CHB	5335	304	276	478			3412	168	226	322		
JPT	5328	311	267	487			3410	180	210	325		
YRI	6093	393	255	574			3692	202	211	364		
All Pop Union	8233	909	703	1355	587	1502	4518	484	560	863	461	973

asc: associated
DE: differentially expressed
