# Supplementary material for: Modifier Effects between Regulatory and Protein-Coding Variation
Source: PLoS Genet. 2008 Oct 31;4(10):e1000244. doi: 10.1371/journal.pgen.1000244 (PMC2570624; doi:10.1371/journal.pgen.1000244)
Supplement: Table S2 — Differentially expressed nsSNPs in OMIM. (0.02 MB PDF) [file pgen.1000244.s003.pdf]

Table S2

Gene	Gene role	Health impact	OMIM ID	DE nsSNPs	Variant role
ABCA1	cholesterol efflux pump in cellular lipid removal pathway	cholesterol transport, familial hypercholesterolemia	600046	rs2230806	Protection against coronary heart disease in familial hypercholesterolemia
				rs28933692	High density lipoprotein cholesterol deficiency (nsSNP role unclear)
				rs28937313	Tangier disease (nsSNP role unclear)
				rs28937314	Tangier disease (nsSNP role unclear)
ADAR	RNA modifying activity	pigmentation	601059	rs28936680	Dyschromatosis symmetrica hereditaria (nsSNP role unclear)
				rs28936681	Dyschromatosis symmetrica hereditaria (nsSNP role unclear)
ADRB2	beta adrenergic receptor	asthma, obesity, vasoconstriction, heart failure	109690	rs1042713	Susceptibility to nocturnal asthma (nsSNP role unclear)
				rs1042714	Significant association with obesity susceptibility
				rs1800888	Profound reduction in sensitivity to vasodilation, vasoconstrictor sensitivity increased
AKAP10	A-kinase anchoring	longevity	604694	rs203462	Singificant difference in frequency between young and old individuals, associated with a negative impact in health and therefore longevity
ALG12	asparigine glycosylation	glycosylation disorder	607144	rs28942090	Hypoglycosylation of serum transferrin
APRT	purine nucleotide salvage pathway	APRT deficiency	102600	rs28999113	APRT abnormal kinetics and low activity (nsSNP role unclear)
ARSA	lysosomal enzyme	lysosomal storage disease affecting growth and development of myelin	607574	rs2071421	Enzyme activity or stability not affected
				rs28940893	Juvenile metachromatic leukodystrophy (nsSNP role unclear)
				rs28940894	Adult metachromatic leukodystrophy (nsSNP role unclear)
				rs28940895	Adult metachromatic leukodystrophy (nsSNP role unclear)
BARD1	BRCA1 interaction	breast cancer susceptibility	601593	rs28997576	Breast cancer-predisposing allele. nsSNP occurs in region controlling growth suppression and apoptosis
BBS2		Bardet-Biedl Syndrome 2	606151	rs4784677	nsSNP role unclear
BCS1L	mitochondrial respiratory chain assembly	Gracile syndrome, iron metabolism	603647	rs28937590	nsSNP role unclear
BRCA1	tumour suppressor	breast/ovarian cancer	113705	rs1800709	Common mutation with moderate phenotype
				rs28897672	nsSNP role unclear
				rs4986852	nsSNP role unclear
BRCA2	tumour suppressor	breast cancer	600185	rs144848	nsSNP role unclear
C10ORF2	mitochondrial protein	Ophthalmoplegia	606075	rs28937887	nsSNP role unclear
CDKN1A/p21	cell cycle control	Tumour develpoment	116899	rs1801270	nsSNP role unclear
CLN5	lysosomal protein (putative)	neuronal ceroid lipofuscinosis	608102	rs28940280	nsSNP role unclear
CTH	cysteine metabolism	elevated homocysteine	607657	rs1021737	Significantly higher concentrations of plasma total homocysteine in isoleucine homozygotes
CTSC	lysosomal protease	periodontitis	602365	rs28937571	nsSNP role unclear
CYP1B1	mixed-function monooxygenase (putative)	primary congenital glaucoma	601771	rs28936700	nsSNP role unclear
				rs9282671	nsSNP role unclear
				rs28936701	nsSNP role unclear
DLG5	epithelial cell structure and signalling	Crohn disease	604090	rs1248696	Susceptibility to Crohn disease (nsSNP role unclear)
DPYD	uracil and thymidine catabolism	DPYD deficiency	274270	rs1801265	nsSNP role unclear
				rs1801267	nsSNP role unclear
DSP	epithelial cell intercellular junctions	Skin fragility - wooly hair syndrome	125647	rs28931610	Severe keratoderma. Substitution of a cysteine is predicted to affect intrachain/interchain disulfide bonding, thus changing the tertiary structure.
ECGF1	angiogenesis and endothelial cell growth stimulation	mitochondrial neurogastrointestinal encephalomyopathy syndrome	131222	rs28931613	Substitution of a positively charged by an uncharged amino acid may account for loss of enzyme activity
EPHX2	detoxication	familial hypercholesterolemia	132811	rs751141	Modifies familial hypercholesterolemia phenotype in individuals with defective low density lipoprotein receptor (LDLR)
GAA	glycogen degredation		606800	rs1800309	nsSNP role unclear
GGCX	modification of vitamin K-dependent proteins	vitamin K-dependent coagulation defect	137167	rs28928872	Homozygote state led to deficiency of all vitamin K-dependent coagulation factors
GHRL	growth hormone regulation	obesity	605353	rs4684677	Obesity susceptibility, nonconservative amino acid change significantly higher in obese children. (nsSNP role unclear)
				rs696217	Obesity age of onset (nsSNP role unclear)
HBG2	fetal hemoglobin	Hb Waynesboro, Hb Sacromonte, Hb Calabria	142250	rs1061234	nsSNP role unclear
				rs28933078	nsSNP role unclear
				rs28933080	nsSNP role unclear
HRAS	oncogene	thyroid carcinoma	190020	rs28933406	nsSNP role unclear
KCNA1	potassium channel	myokymia, ataxia, epilepsy	176260	rs28933381	nsSNP role unclear
				rs28933382	nsSNP role unclear
				rs28933383	Substitution ocurrs in highly conserved position of potassium channel and is predicted to impair neuronal repolarization.
LEPR	adipose tissue mass regulation	glucose response	601007	rs1137100	Impaired glucose response (nsSNP role unclear)
				rs1137101	Differences in body mass index, fat mass, and serum leptin levels (nsSNP role unclear)
				rs8179183	Association with impaired glucose tolerance (nsSNP role unclear)
NPC1	similarity to morphogen receptor "patched"	Niemann-Pick disease type C1	607623	rs28940897	nsSNP role unclear
				rs28942105	nsSNP role unclear
				rs28942106	nsSNP role unclear
				rs28942108	nsSNP role unclear
NR2E3	retinal nuclear receptor	enhanced S-cone syndrome.	604485	rs28937873	nsSNP role unclear
OAS1	resistance to viral infection (possible role in cell growth, differentiation and apoptosis)	diabetes	164350	rs3741981	Association with type 1 diabetes
P2RX7	cell surface ATP receptor	chronic lymphatic leukemia	602566	rs28937574	Susceptibility to chronic lymphatic leukemia. Loss of function mutation
				rs3751143	Susceptibility to chronic lymphatic leukemia. Loss of function mutation
PI	protease inhibitor	emphysema, liver disease	107400	rs11558261	Increased risk of emphysema and liver disease (nsSNP role unclear)
				rs1802959	Increased risk of emphysema and liver disease (nsSNP role unclear)
				rs28929471	Rare 'normal' allele (nsSNP role unclear)
				rs28929473	High risk of emphysema (nsSNP role unclear)
				rs28929474	Deficient PI (nsSNP role unclear)
				rs28931568	Causes Alpha-1 antitrypsin deficiency and emphysema
				rs28931569	Reduced catalytic activity, instability, low plasma concentration. Homozygotes have a high risk of emphysema
				rs28931570	Mildly increased risk of emphysema (nsSNP role unclear)
				rs28931572	Increased risk of emphysema and liver disease. Substitution of polar for nonpolar amino acid predicted to distrupt tertiary structure
				rs709932	nsSNP role unclear
SCO2	cytochrome c oxidase synthesis	infantile cardioencephalomyopathy	604272	rs28937598	cytochrome c oxidase deficiency (nsSNP role unclear)
				rs28937868	cytochrome c oxidase deficiency (nsSNP role unclear)
